# Hemothorax and Pneumothorax Secondary to Costal Involvement in Hereditary Multiple Exostoses: A Systematic Review of Reported Cases in the Literature

**DOI:** 10.7759/cureus.16326

**Published:** 2021-07-12

**Authors:** Kristin Sheaffer, Sarah Hampton, Emily Barnard, Meet N Patel, Lucas Kim, Julian L Gendreau

**Affiliations:** 1 School of Medicine, Mercer University, Savannah, USA; 2 Biomedical Engineering, Johns Hopkins University, Baltimore, USA

**Keywords:** hereditary multiple exostoses, costal exostosis, osteochondroma, rib, hemothorax, pneumothorax

## Abstract

Hereditary multiple exostoses (HME) are an autosomal dominant skeletal disorder characterized by the development of multiple benign osteochondromas (exostoses) that frequently involve long bones of the body. Less commonly, the ribs are a site of involvement, and long-term friction between an exostosis and pleura can produce a hemothorax or pneumothorax. The purpose of this study is to provide a comprehensive review of existing literature on pneumothorax or hemothorax secondary to costal exostosis in HME patients. We reviewed the databases of PubMed and Embase and included data as current as of February 15, 2021. All case reports included cases of hemothorax or pneumothorax in patients with a known personal or family history of HME. After evaluation for inclusion based on eligibility criteria, 18 cases were included. The average age at presentation was 11.7 years (range: 3-32), and most patients were male (83%). Hemothoraces occurred in 15 cases, while pneumothoraces occurred in three cases. All cases were evaluated using chest X-ray and CT scan, and the majority of the cases were treated with surgical resection of the exostosis, either with video-assisted thoracoscopic surgery (VATS; 61%) or thoracotomy (22%). Outcomes were successful with no cases of recurrence after surgical intervention. Although rare, costal exostosis should be considered as a differential in patients presenting with pneumothorax or hemothorax and past medical history or physical exam findings suggestive of HME. Immediate evaluation and surgical intervention to resect costal exostosis are essential to reduce the risk of recurrent life-threatening injury.

## Introduction and background

Hereditary multiple exostoses (HME) is an autosomal dominant skeletal disorder characterized by the development of multiple benign osteochondromas or exostoses. Linkage analysis has revealed that mutations in the EXT family of genes are implicated in the abnormal proliferation of chondrocytes at sites of exostosis formation [1,2]. HME is typically diagnosed in the first decade of life as osteochondromas gradually enlarge until skeletal maturity is reached after puberty. The most common sites of exostosis formation involve long bones, such as the femur (66-90%), tibia (64-84%), and humerus (72%), which can result in skeletal deformities, pain, and compression of neurologic or vascular structures [2,3]. Less clinically apparent is rib involvement, which in the majority of patients presents asymptomatically despite arising in 31-44% of those with HME [3-5]. Symptomatic rib exostosis is rare but its sequelae can result in life-threatening pneumothorax or hemothorax. According to the literature, there have been less than 30 cases reported in this population. Due to the rarity of these events, the primary aim of this study is to provide a thorough review of the existing literature on hemothorax and pneumothorax secondary to costal exostosis in HME patients.

This article was previously presented virtually as a poster presentation at the Mercer University 2021 Annual Joint Research Conference on May 13, 2021. 

## Review

Methods

Search Strategies and Information Sources

A systemic review of the literature was conducted according to the guidelines set by PRISMA (Preferred Reporting Items for Systemic Reviews and Meta-Analyses) [6]. A literature search was conducted using the databases of PubMed and Embase up until February 15, 2021. The following search terms were used in all fields for all databases: ("hereditary multiple exostoses" or “hereditary multiple osteochondromas") and ("pneumothorax”, “hemothorax”, or “hemopneumothorax”). Articles found with this search were screened according to the selection criteria by two authors (KS, SH) for their suitability of inclusion; any disagreements were resolved by consensus among all authors of the study. References of these qualifying articles were also screened for potential inclusion. 

Eligibility Criteria

Abstracts of all case reports were screened to ensure sufficient information was present for review. Case reports considered to be eligible included patients with a stated known personal or family history of HME that had experienced a pneumothorax, hemothorax, or hemopneumothorax secondary to costal exostosis. Literature that was not in the English language, not published in a peer-reviewed journal, or with unobtainable full text was excluded.

Data Extraction

All cases identified via PubMed and Embase were analyzed individually by the authors. Data were collected from text, tables, figures, and graphs provided in the articles. Required parameters and characteristics of each study were extracted and included author names, year, study nationality, demographics of the cases (age, gender), site of exostoses, imaging modalities utilized, treatment, and patient outcomes. In the event of any disagreement with decisions regarding data extraction, a consensus was made among all the authors. All case reports included in this systematic review were thoroughly examined and assessed for the possibility of bias in reporting case data.

Statistical Analysis

Data collection was performed using Microsoft Excel 2020 version 16.45 (Microsoft Corp, Redmond, Washington). Quantitative variables were expressed as either mean or median.

Results

Study Selection

**Figure 1:**
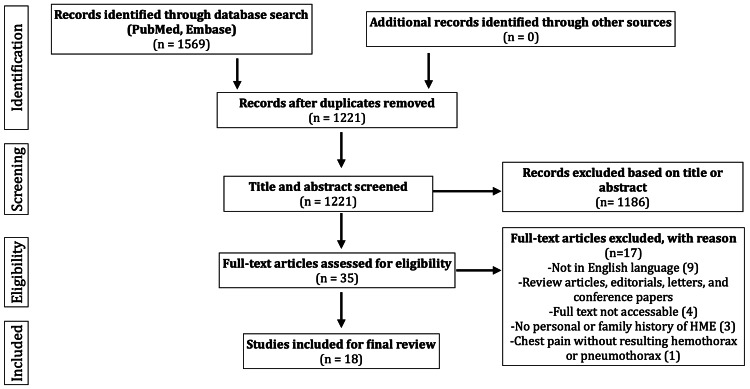
PRISMA flow diagram illustrating the number of articles excluded at different stages of the screening process PRISMA: preferred reporting items for systematic reviews and meta-analyses.

The database search through PubMed and Embase generated a total of 1569 results. After removing 348 duplicate studies, 1221 articles remained to be screened for eligibility. After the additional screening of titles and abstracts, a total of 35 case reports and case series about potential HME patients who had experienced pneumothorax or hemothorax remained. Articles were then further assessed for inclusion criteria by full-text screening. After applying exclusion criteria, 18 case reports remained which met eligibility criteria established by the authors [7-24]. The PRISMA flow diagram is displayed in Figure 1.

Description of Studies

Data from a total of 18 case reports of HME patients with pneumothorax or hemothorax secondary to costal exostosis were obtained. The mean age of the patients was 11.7 years (range: 3-32). The majority of patients were male (83%), which is inconsistent with the lack of gender predominance in HME [2,5]. A total of three cases of pneumothorax and 15 cases of hemothorax in this patient population were reported. Patient demographics including mean age, gender, and presentation characteristics are provided in Table 1.

**Table 1:** Demographics and clinical presentation of costal exostosis as a secondary cause of hemothorax and pneumothorax

Age mean in years (range)	11.7 (3-32)
Gender	n (%)
Male	15 (83%)
Female	3 (17%)
Presentation	n (%)
Pneumothorax	3 (17%)
Hemothorax	15 (83%)

Presentation and Imaging Studies

All patients with both hemothorax and pneumothorax were symptomatic at presentation. The most common symptoms were chest pain (94%) and shortness of breath (39%). All patients were initially evaluated by chest X-ray, followed by CT, which was used to assist in surgical planning and help identify the additional diaphragmatic damage that was present in 36.3% of cases [10,15,16,19]. The most common reported sites of exostosis were the right seventh (19.0%), sixth (14.3%), and eighth ribs (14.3%; Table 2).

**Table 2:** Previous cases in the literature of hemothorax and pneumothorax secondary to costal exostosis VATS: video-assisted thoracoscopic surgery.

Case	Age	Sex	Diagnosis	Exostosis location	Treatment
Dumazet et al. [7]	32	M	Left pneumothorax	Left 4^th^ and 5^th^ ribs	Initial conservative management followed by VATS weeks later with partial resection of 4^th^ and 5^th^ ribs.
Chawla et al. [8]	12	M	Left tension pneumothorax	Left 4^th ^rib	Both managed with chest drain; followed with VATS 2 months after right-sided pneumothorax with excision of exostoses bilaterally.
			Right-sided pneumothorax (6 months later)	Right-sided exostosis not described	
Imai et al. [9]	16	M	Right pneumothorax	Right 7^th^ and 8^th^ribs	Chest drain followed by VATS with partial resection of rib exostosis.
Assefa et al. [10]	14	F	Left hemothorax	Left 7^th^ and 9^th^ ribs	VATS with resection of 7^th^ and 9^th^ ribs, diaphragm repair.
Mann et al. [11]	17	M	Left hemothorax	"Several"	VATS without exostosis removal
			Recurrence 4 days later		Recurrence: VATS with rib shaving and chest tube placement.
Cowles et al. [12]	6	F	Left hemothorax and pericardial effusion	Left (not specified)	VATS with excision of three exostoses.
Teijeria et al. [13]	7	M	Left hemothorax and diaphragmatic rupture	Left 6^th^ rib	Thoracotomy with exostosis resection.
Takata et al. [14]	4	M	Left hemothorax	Left 6^th^ rib	Thoracotomy with resection of the exostosis, a segment of the left 6^th^ rib and adjacent pleura.
Yoon et al. [15]	20	M	Right hemothorax	Right 6^th^ rib	VATS with exostosis resection, diaphragm repair.
Lin et al. [16]	12	M	Right hemothorax	Right 6^th^ rib	VATS with partial exostosis resection, diaphragm repair.
Marlowe et al. [17]	10	M	Right hemothorax	Right 7^th^ rib	Drainage catheter and observation; surgical intervention refused.
Castells et al. [18]	19	M	Right hemothorax	Right 7^th^ and 8^th^ribs	Thoracostomy with drain and observation.
Tomos et al. [19]	14	M	Right hemothorax	Right 8^th^ and 9^th ^ribs	Thoracotomy with partial rib resection, diaphragm repair, and prosthetic patching of the chest wall.
Uchida et al. [20]	19	M	Left hemothorax	Left 2^nd^ rib	Thoracotomy with exostosis resection.
Matsuno et al. [21]	3	M	Left hemothorax	Left 7^th^ rib	VATS with exostosis resection.
Tomares et al. [22]	3	M	Right hemothorax	Right 6^th^ rib	Thoracotomy followed by VATS with exostosis removal.
Huang et al. [23]	9	F	Right hemothorax	Right 7^th^ rib	Thoracentesis followed by observation.
Simansky et al. [24]	17	M	Right hemothorax	Right 9^th^ rib	VATS with exostosis removal.

Treatment

Surgical treatment with exostosis removal was the preferred treatment modality. Video-assisted thoracoscopic surgery (VATS) was the most utilized method, used in 11 cases (61%). There were three patients who underwent removal with VATS after their initial discharge, with the longest delay taking place two months after initial presentation [7,8,11]. Thoracotomy was the other major surgical procedure employed in four cases (22%). There were three patients who did not undergo surgical removal; two opted for thoracostomy and drain, and one underwent thoracentesis alone [17-19]. Surgical and non-surgical interventions are displayed in Table 2.

Outcome and Follow-Up

Information regarding patient outcome after treatment was available in 16 of the cases. There was one instance of postoperative atelectasis, but all others who reported an outcome indicated no complications [13]. Follow-up of patients occurred in four cases, with an average follow-up period of 1.69 years (range: 0.25-4 years) [11,15,22,23]. Of these cases, there were no reported recurrences of hemothorax or pneumothorax during the follow-up period.

Discussion

HME were first described in the literature by John Hunter in his Lectures on the principles of surgery in 1786 [3]. HME is an autosomal dominant skeletal disorder characterized by the development of multiple benign osteochondromas at the juxta-epiphyseal region of long bones [2-4]. Linkage analysis has demonstrated that the *EXT1* (8q24.1) and *EXT2* (11p13) genes are most strongly associated with HME [1]. Mutations in *EXT1* and *EXT2* are theorized to impair heparan sulfate proteoglycan synthesis, leading to abnormal chondrocyte proliferation at sites of exostosis formation [1]. The most common locations for exostoses include the femur, tibia, fibula, ulna, and radius. Less commonly involved are the foot, scapula, hand, ribs, pelvis, and clavicles are involved [1]. These lesions most commonly arise within the first decade of life and enlarge until puberty, which is consistent with the mean age of our study population (11.7 years) [4]. More often than not, exostoses are asymptomatic. For those that are symptomatic, complaints center around pain, deformity, short stature, limb-length discrepancies, bowing of the extremities, or nerve and vascular compression [2,4]. Malignant transformation of benign osteochondromas to chondrosarcomas is also a significant complication of HME, reported in 0.5-10% of patients [2,25].

Particularly, costal exostoses are described between 35% and 44% of HME cases, depending on *EXT1* or *EXT2* genotype [3-5]. They tend to remain asymptomatic or have complaints of atypical chest pain [26]. Rarely, patients with costal exostosis can present with major intrathoracic complications such as pneumothorax, hemothorax, pericardial or diaphragmatic injuries [7-24]. In our literature review, there were three reported cases of pneumothorax and 15 cases of hemothorax in patients with a confirmed personal or family history of HME. Chawla et al. reported a particularly unique case where the patient presented with a second pneumothorax of the contralateral side six months after initial treatment [8].

A few etiological mechanisms of how costal exostosis can cause pneumothorax and hemothorax have been proposed. Long-term friction between exostosis and visceral pleura may be the major contributor to a pneumothorax [9]. For hemothorax, Lin et al. proposed a similar mechanism, where sharp edges of osteochondromas cause repetitive trauma to the diaphragm and pleura during the respiratory cycle [16]. Nevertheless, the clinical presentation of pneumothorax and hemothorax in HME patients parallels that of a standard patient. In our review, all but one patient presented with acute onset chest pain (94%), and in some this was the only reported symptom. The most common other symptoms were dyspnea (38%) and abdominal pain (11%), likely secondary to diaphragmatic irritation [12,13].

Diagnostically, all cases were first evaluated with chest X-rays due to their clinical symptoms. Although chest X-ray is standard for initial evaluation of pneumothorax or pleural effusion, it can be difficult to visualize the exact location of exostoses in relation to surrounding structures [27]. After initial pathology was identified on chest X-ray, a chest CT scan was ordered. Chest CT scan is the standard for emergent complications of costal HME and allows for ease of immediate surgical planning [27]. From imaging, three cases of pneumothorax (17%) and 15 cases of hemothorax (83%) were diagnosed. The ratio of right-to-left-sided involvement was 1:1, and the most commonly reported sites of costal exostosis that resulted in intrathoracic trauma were the right seventh (19.0%), sixth (14.3%), and eighth ribs (14.3%; Table 2).

Surgical intervention with resection of exostosis is the most common method of treatment for symptomatic costal exostosis. Additional indications for surgical removal of costal exostosis include alleviating pain, confirming the diagnosis of benign osteochondroma, or removing malignancy [28]. Removal is most commonly done via VATS or thoracotomy [28]. In our review, the most common method of treatment was surgical removal via resection or shaving down the exostosis with or without partial rib resection, which was done in 83% of cases. The study by Mann et al. was the only case that reported shaving the exostosis down flat with the underlying rib; it was not discussed why this was done compared to a resection [11]. In pre-pubertal patients, there is approximately a 2% risk for recurrence after surgical removal [29]. Despite this small chance of recurrence, surgical intervention remains the treatment of choice in symptomatic patients, as watchful waiting without resection carries the risk of additional intrathoracic trauma [11,12,24].

Non-surgical management of pneumothorax or hemothorax in HME patients is also an option and is often used prior to surgical interventions. The most common non-surgical technique utilized was thoracentesis, which the majority of patients (67%) received. Of those patients, all but two underwent a subsequent surgical procedure. In one patient, thoracentesis with conservative follow-up was the only treatment recommended, unless they experienced any future recurrence [23]. Less common management involved thoracostomy and CT-drainage catheter [17,18]. In Marlowe et al., the patient was treated with thoracostomy alone due to refusal of surgical interventions by the patient’s parent, but the authors did recommend VATS or thoracotomy in case of recurrence [17].

The overall outcome of this rare condition appears to be acceptable. The isolated case of same-side recurrence occurred in the hemothorax reported by Mann et al., where the patient underwent diagnostic VATS without exostosis removal [11]. In this case, the patient was discharged, and four days later returned with ipsilateral recurrent hemothorax that was subsequently treated using VATS and the lesion was shaved flat against the rib [11]. Chawla et al. was the case in which the patient developed contralateral pneumothorax six months after initial conservative treatment with a chest drain; he subsequently underwent VATS with removal two months later [8]. One case did develop postoperative atelectasis which required bronchoscopies and intense respiratory therapy prior to discharge [13]. All case reports reviewed otherwise indicated that there were no complications postoperatively, and all patients followed up after discharge had no recurrence of pneumothorax or hemothorax [11,15,22,23].

Limitations

This review has several limitations. First, reported details were not standardized across all case reports, which limits the breadth of data that can be analyzed. This is especially important in this rare presentation of this disease, as only 18 patients are represented in the data. In the cases examined, 83% of patients were male, which is inconsistent with the equal male-to-female ratio seen in HME and introduces the potential for gender bias. Length of follow-up was often not included, and this limits the ability to assess for potential long-term benefits and risks of surgical versus non-surgical intervention for symptomatic costal exostosis in this patient population. Additionally, as our findings were derived from case reports, which rank lowest on the pyramid of evidence, therefore, there is the inherent inability to draw specific conclusions about patient outcomes in regard to recovery, recurrence, and long-term complications.

## Conclusions

Pneumothorax and hemothorax secondary to costal exostosis in patients with HME remain a rare, yet potentially life-threatening phenomenon. Evaluation of these patients should be done with an initial chest X-ray and chest CT scan. Prompt surgical intervention either via VATS or thoracotomy is the most common and most recommended method of definitive treatment. Non-surgical treatment via thoracentesis with or without thoracostomy and drain placement is an option, but this method does not reduce the risk of life-threatening intrathoracic injury. Close follow-up is encouraged to assess for recurrence of exostosis, pneumothorax, or hemothorax, especially in the skeletally immature, and should be considered in future studies regarding exostosis outcome.
